# Disposable plastic trays and their effect on polyether and vinyl polysiloxane impression accuracy—an in vitro study

**DOI:** 10.1007/s00784-020-03455-6

**Published:** 2020-09-03

**Authors:** Stefan Rues, Thomas Stober, Thomas Bargum, Peter Rammelsberg, Andreas Zenthöfer

**Affiliations:** Department of Prosthodontics, University of Heidelberg, Im Neuenheimer Feld 400, 69120 Heidelberg, Germany

**Keywords:** Accuracy, Impression, Disposable plastic tray, Metal tray, Polyether, Vinyl polysiloxane

## Abstract

**Objectives:**

To evaluate the dimensional accuracy of impressions taken by use of disposable stock plastic trays and to compare performance with that of metal trays.

**Materials and methods:**

From a metallic model incorporating three precision balls and three abutment teeth, one-step dual-phase polyether (PE) and vinyl polysiloxane (VPS) impressions were taken using either metal or disposable plastic trays (*n* = 10 for each of the resulting four test groups). Respective plaster cast scans were aligned with the reference dataset to evaluate global (distance and angle deviations) and local (trueness and precision) accuracy. Analyses of variance (ANOVA) were conducted to determine group differences.

**Results:**

For all impression tray and material combinations, global accuracy was good (mean distance changes < 100 μm) with greatest deviations being observed for distances exceeding one quadrant of the dental arch. In general, distances measured in the plaster casts were too short. Only VPS impressions with plastic trays showed a different behavior with a large percentage of cross-arch distances exceeding the reference value. Mean local accuracy ranged between 6 and 14 μm (trueness), and 6 and 16 μm (precision). On abutment tooth level, metal trays were associated with a significantly better precision (*p* = 0.015).

**Conclusions:**

The observed distortions of the studied impression trays and materials are small and should enable satisfying clinical impression-taking.

**Clinical relevance:**

Cleaning and processing of metal trays before re-use are time-consuming. Especially for patients’ management with single crowns and small fixed dental prostheses, disposable plastic trays can be a viable and cost-effective alternative.

## Introduction

In restorative treatment, the reliable communication of the clinical situation to the dental technician is a complex but key process [1]. Nowadays, there are largely two established elastomeric material classes for impression-taking of abutment teeth prepared for fixed and removal dental prostheses: polyether (PE) and vinyl polysiloxane (VPS) [2–5]. Both enable excellent dimensional accuracy and the detail reproduction needed but the material classes also include advantages/disadvantages; just to mention a few, PE materials are designed to show initially hydrophilic behavior which should enable a better flow onto moist tooth structures [3, 6], while VPS materials are rather neutral in taste and can be stored over longer time periods [3, 7]. Hereby, each of the advantages of one material is the disadvantages of the other.

To date, some hybrid materials such as silicones with hydrophilic properties are available on the market, which aim to combine the various advantages [5, 8]. For the sake of completeness, digital impressions by use of intraorally three-dimensional scanners should also be mentioned which seem to be—regarding accuracy—comparable if used for the fabrication of single crowns [9–11], but are still disadvantageous to conventional impression-taking when it comes to long-span or full-arch restorations [9]. It should be also acknowledged that laboratory adjustment of restorations including refinement of fit and occlusal contacts is more challenging without having plaster casts [9].

Beyond this, conventional and digital impression-taking are influenced by various clinical parameters and procedures. Oral hygiene and periodontal health and therefore bleeding tendency, the location of the preparation margin, saliva, and the angulation of the abutment tooth, among others, all affect the accuracy of impressions [9, 12, 13]. With respect to procedural aspects, in digital impression-taking, for instance, powdering which is often necessary when glossy surfaces are positioned next to relevant structures can affect the accuracy.

Back to conventional impression-taking, the technique (one-step/two-step and monophase/dual-phase) can impact the outcome as well as disinfection measures applied to cured impressions [8, 14–18]. A major impact is also attributed to the type and design of the impression trays used. For final impressions, metal trays with flanged margins (border-lock) are recommended as they offer torsional rigidity and inherent stability [19, 20]. The border-lock design combined with the use of tray adhesives hinders detachment of the cured impression. On the other hand, cleaning and processing for re-use are expensive and time-consuming. In times of aggravated hygiene guidelines and centralized sterilization, single-use products grow in importance [21]. As processing steps are omitted, significantly more waste is produced. Nonetheless, disposable plastic impression trays have been introduced to the market and are very attractive in countries with high personnel costs since time for cleaning and sterilization can be saved. Vice versa, there are concerns about the reduced accuracy of impression-taking in this way due to reduced torsional rigidity [19, 20] but comparable accuracy of metal and stock plastic trays has also been presented in the literature [7]. A dental company introduced disposable stock plastic trays with a border-lock design and adhesive fleeces; no information on the accuracy is available in the scientific literature.

The objective of this laboratory study, therefore, was to evaluate the dimensional accuracy and precision of VPS and PE impressions taken by use of these plastic trays in comparison to impressions made with metal border-lock trays. The study hypothesis was that impressions made by the use of the disposable trays come along with reduced accuracy and precision compared with those of the metal trays.

## Material and methods

### Pre-test

To address the concern that with the use of a metallic reference model demolding forces might differ distinctly from the clinical situation, a pre-test was conducted to evaluate the forces needed to demold impressions of natural teeth and metal teeth. A natural molar was prepared in full crown design with a chamfer finishing line. In addition, the test tooth was duplicated in unprepared condition as well as after preparation and reproduced in cobalt-chromium (CoCr) alloy (Remanium Star; Dentaurum, Germany). The four specimens differing in tooth material (natural tooth vs. metallic tooth) and tooth state (before preparation vs. after preparation) were molded in specimen holders in exactly the same position and orientation using autopolymerizing resin (Technovit, Heraeus Kulzer, Germany). To simulate the impression tray, a standardized hollow cylinder of steel with 20 mm of inner diameter was used. The respective specimen was mounted upside down on the upper fixation of a universal testing device (Z005, Zwick/Roell, Ulm, Germany) and coated with low viscous impression material from a hand dispenser after the hollow cylinder filled with tray impression material was placed on the lower fixation. After that, the specimen was lowered into the tray material with a cross head speed of 120 mm/min up to a predefined final position. For each test group, removal forces for two subgroups (*n* = 10 impressions per subgroup) with one-step dual-phase impressions differing in impression material (PE: Impregum Penta Duo Soft/Garant L Duo Soft vs. VPS: Imprint 4 Penta Super Quick heavy/light; 3M Oral Care, Seefeld, Germany) were determined. After the doubled setting time (shrinkage forces were prevented by regular relaxation) of the impression materials (PE: 12 min, VPS: 5 min)—to meet the prolonged setting at room temperature—impressions were demolded with a crosshead speed of 10 mm/min.

### Main trial on impressions’ accuracy

The impact of the impression tray selection, i.e., conventional border-lock metal trays (Ergolock, Size: XL; Omnident, Germany) versus disposable stock plastic trays (Position Tray, Size: L; 3M Oral Care, Seefeld, Germany), on the accuracy was investigated. For each tray group, impressions were taken with both a VPS (Imprint 4 Penta Super Quick heavy/light; 3M Oral Care, Seefeld, Germany) and a PE (Impregum Penta Soft/Impregum Garant Duo Soft; 3M Oral Care, Seefeld, Germany) material processed in one-step dual-phase technique. The accuracy was studied indirectly by measuring dimensional changes of the plaster casts generated by the respective impressions related to a master model.

**Fig. 1:**
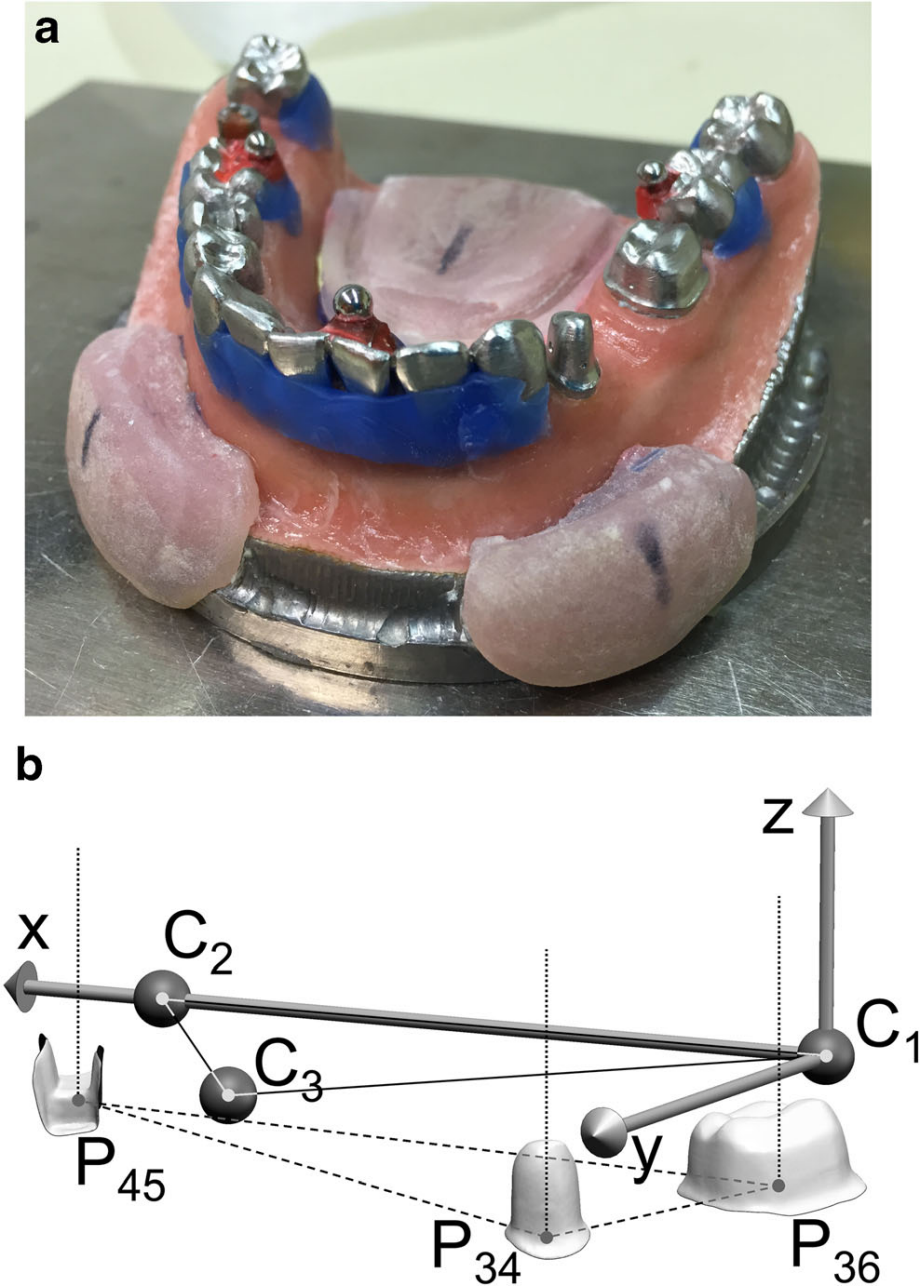
**a** Metallic master model with rest positions for the tray. Deep undercuts were blocked out to avoid very high removal forces. **b** Virtual master model with indicated reference distances and vertical tooth axes

The master model containing CoCr teeth simulated a clinical case with abutment teeth 34 and 36 prepared for the incorporation of a small fixed dental prosthesis and tooth 45 present with an inlay preparation. Additionally, three stainless steel precision balls (quality G3) with 3175 μm diameter were welded to the occlusal aspects of teeth 37 and 46 as well as in the area of the incisal point of teeth 31/41 (cf. Fig. 1a). In the vestibular area of the model, acrylic positioning aids were located, enabling a standardized placement of the impression trays during impression-taking (at least 2 mm space for the impression material to dentition and steel balls). The single reference dies were digitized by a specialized industrial company using an optical profilometer (μscan custom with CF4 sensor, NanoFocus AF) to obtain a highly accurate digital reference dataset (measurement error less than 1 μm) of the respective prepared tooth surfaces. To gain information about the spatial position of the dies and precision balls after welding to the master model, supplementary measurements using a coordinate measuring machine (MarVision MS 222, Hexagon Metrology, about 200 measurement points for each tooth or precision ball, accuracy < 2 μm) were carried out. Both datasets were aligned and the achieved final global coordinate system was defined by the centers of the precision balls (C_1_ in tooth region 37, C_2_ in tooth region 46, and C_3_ in tooth region 31/41) as follows (cf. Fig. 1b):

- Origin of the coordinate system located at C_1_
- x-axis directed along the vector $\overrightarrow{{\mathrm{C}}_1{\mathrm{C}}_2}$
- xy-plane defined by the points C_1_, C_2_, and C_3_
- y-axis in anterior direction

To be able to evaluate distance changes and angular changes between each two prepared teeth, respective local coordinate systems were defined at the centers of the preparation margins (P_34_, P_36_, P_45_) with axes parallel to those of the global coordinate system.

**Fig. 2:**
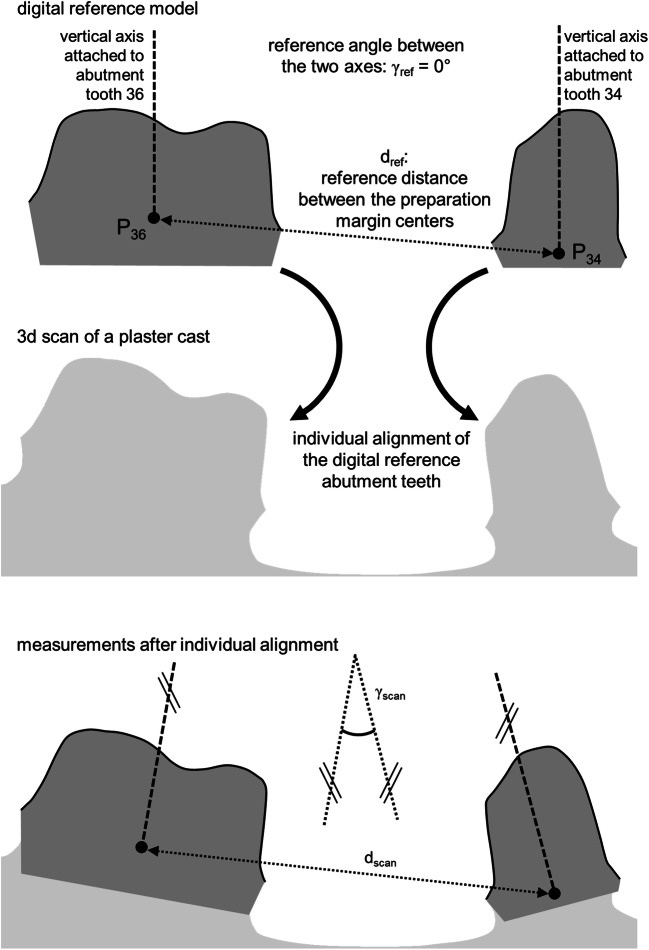
Measurement procedure for distance and angular changes between the abutment teeth exemplarily illustrated for abutment teeth in positions 34 and 36

Reference distances in the master model (between precision ball centers and between preparation margin centers) amounted to C_1_–C_2_: 40.338 mm, C_1_–C_3_: 35.916 mm, C_2_–C_3_: 31.927 mm, P_34_–P_36_: 15.400 mm, P_34_–P_45_: 36.578 mm, and P_36_–P_45_: 41.515 mm. All corresponding axes defined in the master model were parallel; consequently, angles between respective axes of any two teeth were zero (cf. Fig. 2 top)

### Test series

**Fig. 3:**
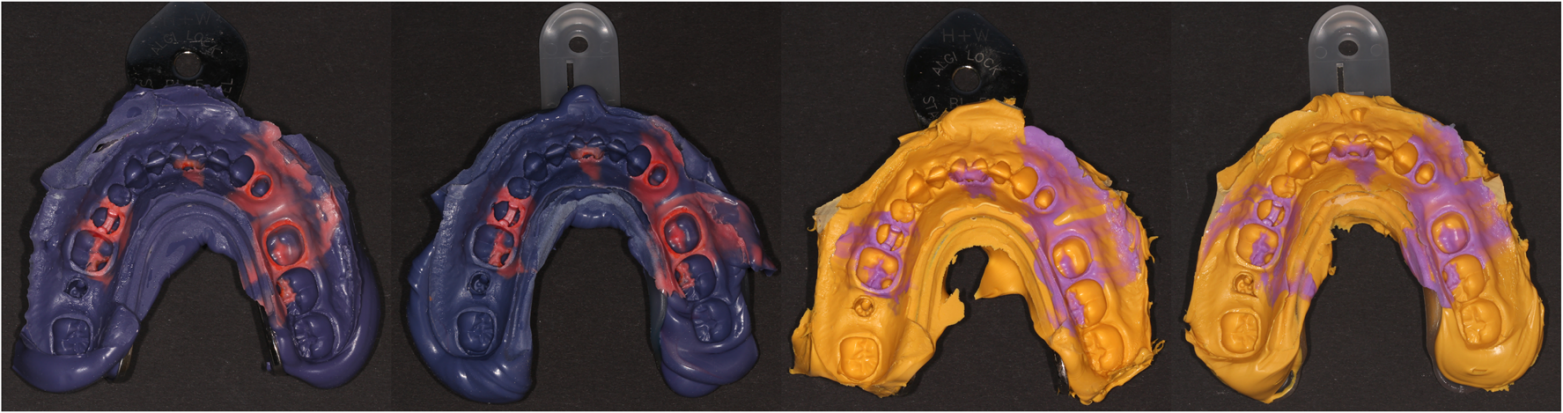
Impressions of the four test groups differing in tray type (metal/plastic) and impression material (PE/VPS)

Each ten impressions in one-step dual-phase technique were taken for the four study groups differing in impression material and tray type (cf. Fig. 3). For the metal tray groups, the respective tray adhesive was used. The disposable plastic trays included retention fleeces on the inner surface of the trays. The setting time of the materials was twice the clinical working time, i.e., 12 min for PE impressions and 5 min for VPS impressions. All impressions taken were stored for 5 min in a disinfection solution (Printosept ID (Alpro, Germany)) and were poured with type IV dental stone (Zero Stone; Dentona AG, Germany) with 0% expansion (information provided by the manufacturer) after a minimum reset time of 60 min.

### Fabrication and digitization of the stone models

Saw-cut models were fabricated using Giroform base plates (Amann Girrbach, Pforzheim, Germany). In case of imperfections in relevant areas, the sample was excluded and reproduced starting with a new impression. To allow a standardized orientation with three-dimensional scanning, a secondary plate (which would be used to mount saw-cut models in an articulator) was adhesively attached to the magnetic interface plate of the 3D scanner (D800; 3Shape, Copenhagen, Denmark). For all specimens, each three series with each six circumferentially distributed scans (single scan resolution: 70 μm) with tilts of 15°, 50°, and 70° with respect to the horizontal plane was performed (Convince 2012, 3shape) and a rather fine and homogeneous mesh generated (triangulation parameters: detail accuracy 9/10, threshold for noise filtering 1/10, iteration optimization of mesh: deactivated). Each stone model was placed and digitized three times.

### Evaluation of deviations between the master model and the plaster casts

Deviations between the digitized master model and the stone model scans were analyzed with the aid of Geomagic Design X (3D Systems, Germany) and Matlab R2015 (Mathworks, Natick, MA, USA). Scan regions showing the precision balls were cropped, sphere center positions calculated by means of optimization (least squares, fixed nominal sphere radius), and sphere distances determined. After use of a coordinate transformation aligning the global scan coordinate system with that of the reference model, each of the three reference surfaces was separately aligned with each scan and the local coordinate systems moved along (cf. Fig. 2). In detail, the following measurement data (*n* = 10 samples per subgroup, 3 measurement repetitions per sample) were reported in this manuscript:

- Global accuracy
  - Distance changes between precision ball centers
  - Distance changes between preparation margin centers and angular changes between the tooth axes
- Local accuracy
  - For each prepared surface, trueness and precision were analyzed separately (based on absolute values) by means of mesh deviation.

### Statistical evaluation

Statistical evaluation was performed with the aid of SPSS Ver. 25 (IBM Corp., New York, USA) at a significance level of *α* = 0.05.

For investigate differences in demolding forces, *t* tests were performed for data differing in impression material. For data associated with each of the two impression materials, an analysis of variance was performed to identify the influence of the “tooth material” and “tooth preparation” factors.

For all test groups, mean values and standard deviations (SD) were computed for data originating from accuracy measurements. In addition, box-plots were used to visualize results where helpful. Analyses of variance and post hoc Tuckey tests were conducted to determine the effect of the “tray material,” “impression material,” and “measurement location” factors (global accuracy: each 3 different reference distances between the precision balls and between the prepared teeth; local accuracy: 3 different prepared teeth) on the accuracy of the generated plaster casts.

## Results

### Retentive forces of the impression materials (pre-test)

**Fig. 4:**
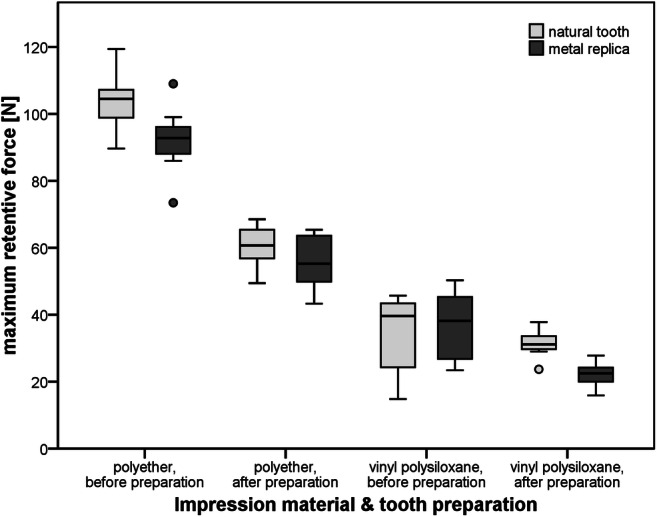
Results of the demolding forces of a single molar depending on impression material, tooth material, and preparation

All results of the retention tests are summarized in the box-plot shown in Fig. 4. With respect to the tooth material (natural tooth vs. metal replica), only small differences in maximum forces needed to demold the impressions were detected. For each of the test groups differing in impression material and tooth preparation, the median values of the demolding forces were slightly lower for the metal replica when compared with those found for the natural tooth. These differences were only significant (*p* = 0.122 for VPS impressions, *p* = 0.002 for PE impressions) for PE impressions. From a clinical point of view, the results indicate that the laboratory setting with a metallic master model did not lead to uncharacteristically high demolding forces. Prepared teeth showed significantly lower retentive forces compared with unprepared teeth (*p* < 0.001). Mean maximum retentive forces were significantly associated with the used impression material, manifesting in 2 to 3 times higher demolding forces for PE impressions when compared with VPS impressions (*p* < 0.001).

### Distance changes between the plaster casts and the master model (global accuracy)

**Fig. 5:**
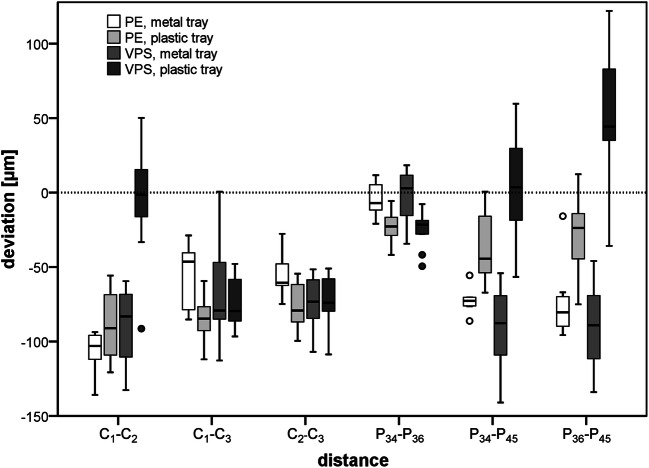
Distance changes between the reference balls C_1_, C_2_, and C_3_ and the preparation margin centers P_34_, P_36_, and P_45_ (global accuracy) of plaster casts generated by the different impression materials/trays (*n* = 10 per group)

The deviations of the respective path lengths measured in the datasets generated by digitization of the plaster casts of the respective impressions are presented in the upper part of Table 1 and depicted in Fig. 5. In relation to the path lengths, mean deviations of the test groups ranged in mean between − 2.5 and − 0.1‰ for the precision balls and − 2.5 and + 1.25‰ for the preparation margin centers. In general, distances were shorter in the plaster casts when compared with the reference values. A high percentage of the measured distances was greater than the reference value only for cross-arch distances (C_1_–C_2_, P_34_–P_45_, P_36_–P_45_) with plaster casts generated from VPS impressions with plastic trays.

**Table 1:** Distance changes between the reference balls C_1_, C_2_, and C_3_ and the preparation margin centers P_34_, P_36_, and P_45_ as well as angular changes between the vertical tooth axes (global accuracy) of plaster casts generated by the different impression materials/trays (*n* = 10 per group). Significant differences are indicated with capital letters for the test groups and with lowercase letters for the measurement location

Distance changes, mean value (standard deviation) (μm)				
Distance between	Polyether (PE)		Vinyl polysiloxane (VPS)	
	Metal tray^A^	Plastic tray^A^	Metal tray^A^	Plastic tray^B^
C_1_–C_2_^a^	− 102 (26)	− 89 (22)	− 90 (26)	− 3 (40)
C_1_–C_3_^a^	− 55 (21)	− 86 (15)	− 67 (38)	− 75 (16)
C_2_–C_3_^a^	− 56 (13)	− 77 (16)	− 74 (17)	− 72 (17)
Distance changes, mean value (standard deviation) (μm)				
Distance between	Polyether PE		Vinyl polysiloxane VPS	
	Metal tray^A,B^	Plastic tray^B^	Metal tray^A^	Plastic tray^C^
P_34_–P_36_^a^	− 5 (11)	− 22 (11)	− 2 (17)	− 25 (12)
P_34_–P_45_^b^	− 67 (20)	− 38 (23)	− 92 (29)	+ 6 (35)
P_36_–P_45_^a,b^	− 75 (23)	− 28 (26)	− 90 (27)	+ 52 (47)
Angular changes between tooth axes, mean value (standard deviation) (°)				
Z-axes of teeth	Polyether (PE)		Vinyl polysiloxane (VPS)	
	Metal tray^A^	Plastic tray^A^	Metal tray^A^	Plastic tray^A^
34, 36^a^	0.16 (0.03)	0.14 (0.04)	0.20 (0.08)	0.20 (0.05)
34, 45^b^	0.79 (0.09)	0.85 (0.11)	0.85 (0.09)	1.01 (0.16)
36, 45^b^	0.82 (0.09)	0.93 (0.13)	0.73 (0.08)	1.18 (0.17)

For the distances between precision balls, the factor “measurement location” did not affect the measured deviations, i.e., similar distance changes were observed for all three spans (*p* = 0.968). Distance changes were significantly lower for VPS impressions using the disposable plastic tray compared with those of the metal tray (*p* < 0.001). All other group comparisons missed the level of statistical significance.

Regarding the distance changes between the reference points located at the preparation margin centers, a higher accuracy was observed for the short span between teeth 34 and 36 compared with the cross-arch distances 34–45 and 36–45 (the factor “measurement location” had a significant influence, *p* < 0.001). Of the teeth 34 and 36, mean deviations between the master model and the plaster casts in mean (SD) ranged between − 2 (17) for the VPS impression material in use with a metal tray and − 25 (12) for VPS used in combination with the disposable plastic tray (see Table 1). The analysis of variance yielded a significant effect on the type of impression tray used with a greater deviation for the plastic tray (*p* < 0.001). Bivariate comparisons showed that both types of impression materials used with plastic trays resulted in greater deviation than in use with metal trays (PE: *p* = 0.002; VPS: *p* = 0.003).

The highest accuracy with respect to angular changes between two tooth axes was observed between abutment teeth 34 and 36 with mean values < 0.2° for all test groups. Here, no effect of the type of tray used could be detected (*p* > 0.05) but significant differences were observed with regard to the factor “impressions material” indicating an advantage of PE over VPS (*p* = 0.01). For the other two tooth pairings (34 and 45, 36 and 45) impression material (*p* < 0.038) and tray type (*p* < 0.004) had a significant influence. The smallest angular deviations were found for plaster casts made from PE impressions with metal trays, whereas the greatest angular changes were associated with plaster casts made from VPS impressions with plastic trays (cf. Table 1, lower part).

### Surface deviations of the prepared teeth (local accuracy)

**Fig. 6:**
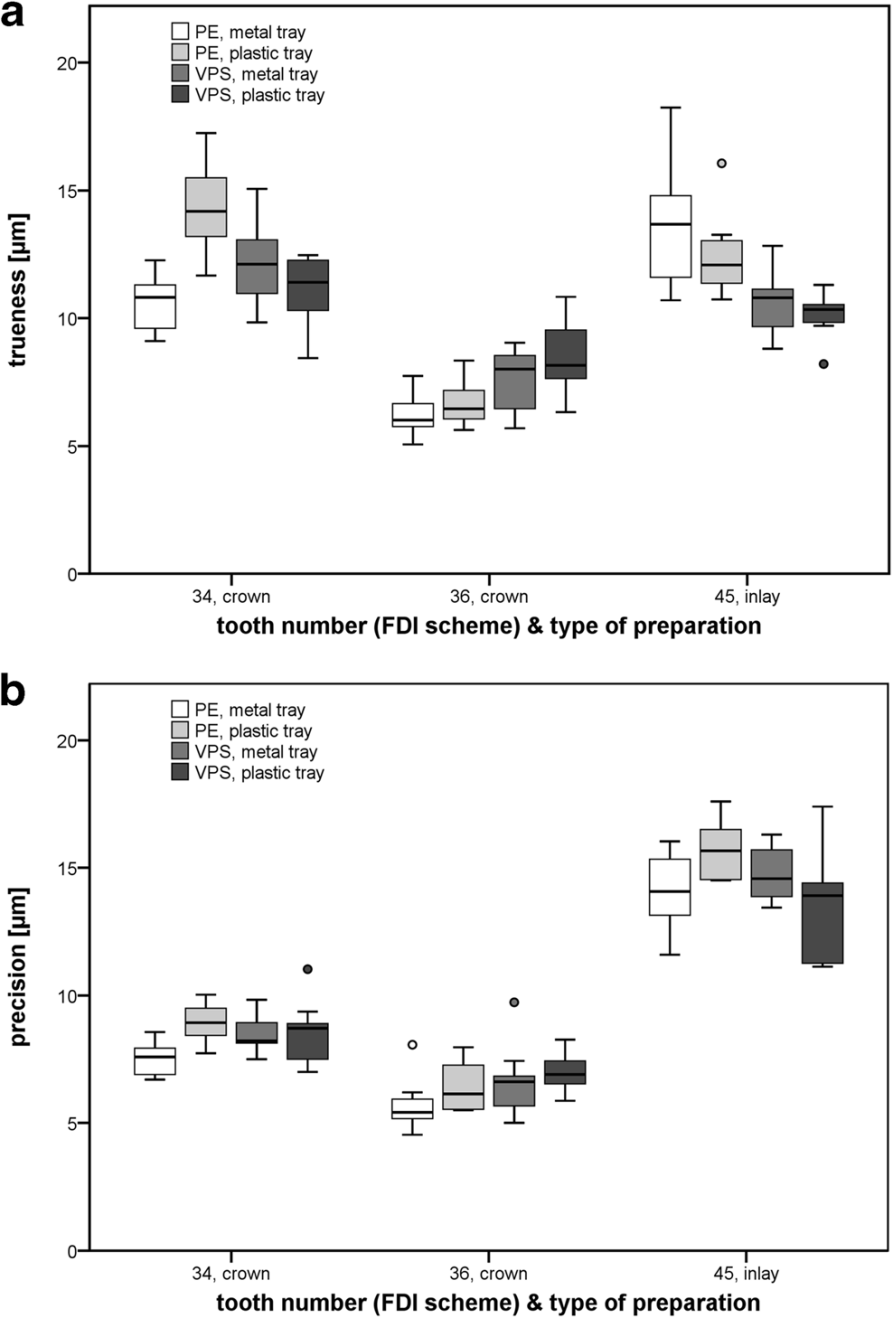
**a** Trueness and **b** precision (local accuracy) of the plaster casts generated by the different impression materials and tray types (*n* = 10 per group), calculated separately for each abutment tooth

Table 2 presents the trueness and precision for the different prepared teeth of the replicas for the impression trays and materials. Mean values for trueness (cf. Fig. 6a) ranged between 6 and 14 μm. Analysis of variance detected a significant effect of the impression material used (*p* = 0.012), while the tray selection only showed a trend (*p* = 0.087) to influence trueness.

**Table 2:** Trueness and precision (local accuracy) of the plaster casts generated by the different impression materials and tray types (*n* = 10 per group), calculated separately for each abutment tooth. Significant differences are indicated with capital letters for the test groups and with lowercase letters for the different abutment teeth

Tooth number (FDI)	Type of preparation	Polyether (PE)		Vinyl polysiloxane (VPS)	
		Metal tray^A^	Plastic tray^A^	Metal tray^A^	Plastic tray^A^
		Trueness, mean value (standard deviation) (μm)			
34^a^	Crown	10.6 (1.1)	14.4 (1.7)	12.2 (1.5)	11.1 (1.4)
36^b^	Crown	6.2 (0.7)	6.7 (0.8)	7.6 (1.1)	8.4 (1.3)
45^a^	Inlay	13.5 (2.2)	12.4 (1.5)	10.5 (1.2)	10.2 (0.9)
		Precision, mean value (standard deviation) (μm)			
34^a^	Crown	7.5 (0.6)	8.9 (0.8)	8.4 (0.7)	8.5 (1.2)
36^b^	Crown	5.7 (1.0)	6.4 (1.0)	6.6 (1.3)	7.1 (0.8)
45^c^	Inlay	14.0 (1.4)	15.6 (1.1)	14.7 (1.1)	13.6 (2.0)

Precision (cf. Fig. 6b) also showed only small group differences (mean values ranging between 6 and16 μm). The statistical effects of tray type and impression material on precision were, however, contrary to those found for trueness: the impression tray used affected precision (*p* = 0.015), whereas no impact regarding the impression material (*p* = 0.557) could be seen.

With regard to abutment tooth and type of preparation, abutment tooth 36 with a crown preparation showed the best accuracy and abutment tooth 45 with an inlay preparation showed the worst accuracy (*p* < 0.001 for both trueness and precision).

## Discussion

The study hypothesis had to be partly rejected. Regarding single tooth, local accuracy was excellent for all impressions and no difference was found between study groups differing in tray type. With respect to global accuracy, the results of this laboratory study suggest that the deviations of the plaster casts generated by use of PE and VPS material combined with the use of disposable plastic and metal trays were in general small (in mean, all smaller than 100 μm on distances up to approximately 4 cm) but partially significantly different. In particular, for the fabrication of single crowns and small fixed dental prostheses, impressions taken by the use of stock plastic trays were not inferior compared with impressions taken with metal trays. With respect to the metal trays, the observed size of deviations is similar to that seen in previous studies on the accuracy of precision impressions [22, 23].

Interestingly, some studied test parameters reflected an even higher accuracy if plastic trays were used. Hence, if VPS was used for impression-taking, mean deviations of measurement paths compared with the master model were significantly smaller in use of plastic trays than for metal trays (horizontal cross-arch span between the reference balls). Furthermore, this was the only test group showing positive deviations (measured distances were larger than the reference). One might speculate that the rather fast-setting VPS had a higher heat production rate compared with the PE which caused a thermal expansion of the plastic tray. A rough estimation using a temperature change of 5 K and a coefficient of thermal expansion of 100 10^−6^ K^−1^ would lead to a thermal strain of 0.5‰ which alone would not be enough to cause the observed changes. Noticeable distortions of the plastic tray due to the shrinkage of impression material may also occur and contribute to the observed effect.

The overall scaled-down situation compared with other studies might be explained by the fact that a plaster with zero expansion was used for fabrication of the replica models and shrinkage of the impression materials was possibly not compensated. Since we did not validate the manufacturer’s information regarding the plaster’s expansion, this assumption has to be taken with care. In previous studies, the accuracy of impression materials was frequently evaluated indirectly via replicas of the master model, too. The direction of deviations of the replicas to be expected from those studies is very inconsistent.

Some authors found enlarged distances if one-step dual-phase elastomeric impression materials are used [14], while others found both enlarged and scaled-down measurement paths [22, 23]. A recent study by Arora et al. found, in accordance with the results presented in this study, replicas to be smaller in dimensions than the master model [24].

Returning to the comparison of the accuracy of impressions done with disposable plastic trays and metal trays, respectively, for the clinical situation of the abutment teeth 34/36 prepared for the incorporation of a small fixed dental prosthesis, a significantly lower accuracy—irrespective of the impression material used—was detected for plastic trays. An early review about the influence of tray selection on the accuracy of impressions stated that flexible plastic trays lead to considerable discrepancies (approximately 200 μm); presented deviations were stated to be substantially higher than was the case with metal trays, indicating that they were not suitable for clinical use if fixed dental prostheses should be fabricated on [18].

Hoyos and Soderholm compared disposable plastic trays and metal border-lock trays regarding their resulting accuracy of putty-wash and one-step dual-phase impression techniques. The authors concluded—in one line with the review—that plastic trays produce less accurate impressions [20]. Contrary to those findings, further investigations did not confirm that outcome [7].

Distortions of impressions done with plastic trays seen in the presented study were in each case substantially lower compared with those in the early review, which fuels speculation on the causes. First, the new generation of stock plastic trays used has a border-lock design and a retentive fleece which keeps the cured impression material in place. Second, they might have a higher torsional rigidity due to more massive cross struts. Third, the specific plastic composition might have a high recovery after demolding similar to that of impression materials. In this context, changes of angles between the prepared to abutment teeth were negligible (< 0.2°) and comparable for impressions done with plastic and metal trays, respectively. Moreover, those angular changes would lie in the tolerance margins of tooth mobility [25]. Specific attention should be paid to this aspect if implant-supported restorations are to be fabricated which have almost no adaptability with regard to angular changes between implant axes [7].

In general, impressions with plastic trays were associated with higher angular changes. Only for the situation of a short fixed partial denture, no significant differences between metal and plastic stock were found. Furthermore, local accuracy (trueness and precision) was superb for both impression materials performed with plastic and metal trays with no significant differences regarding trueness. However, a slight but significant difference in advantage for metal trays compared with plastic trays (precision range 6–16 μm) was seen; however, this difference is not clinically relevant. In fact, taking all aspects of studied accuracy into account, both impression materials used in combination with metal or plastic trays enable clinically satisfying fabrication of at least single crowns or small fixed dental prostheses, keeping in mind the required minimum accuracy for the fit of those restorations to be smaller than 100–150 μm [26, 27]. This study did not investigate the effect of all types of impression materials on possible distortions of the plastic trays leading. Especially for putty impression materials, this issue was not clarified.

### Strengths and weaknesses of the study

The laboratory study design enables a repeatable evaluation of the accuracy of the impression trays and materials. Here, it should be especially emphasized that a pre-test was performed to compare demolding behavior of the impression materials used. However, an in vitro design can never fully capture the clinical reality. Just to mention a few aspects, oral cavity temperature, subgingival preparation, and deviating demolding forces might influence the accuracy of the impression-taking process [12]. An advantage of in vitro investigations is that the geometry of the reference models does not change and can be directly assessed with devices providing a higher accuracy than the 3D scanners used in dentistry. In our study, the optical profilometer and the coordinate measurement machine both provided accuracies less than 2 μm. Acquisition of reference distances in vivo [28], in contrast, is a very challenging problem and can never reach the accuracy level possible with an in vitro model. Plaster itself also comes along with distortion in the clinical situation [29]. However, the authors tried to overcome this problem by the use of zero expansion plaster. Furthermore, a possible bias would influence all test series likewise.

## Conclusions

The observed distortions were small for PE and non-putty VPS impressions performed with the metal and the disposable plastic impression trays. Within the limitations of this laboratory study, plastic trays presented an adequate level of accuracy and are suitable for single crowns and 3-unit fixed dental prostheses. Clinical studies are encouraged to secure the study outcome.
